# Detection and isolation of brain tumors in cancer patients using neural network techniques in MRI images

**DOI:** 10.1038/s41598-024-68567-5

**Published:** 2024-10-07

**Authors:** Mahdi Mir, Zaid Saad Madhi, Ali Hamid AbdulHussein, Mohammed Khodayer Hassan Al Dulaimi, Muath Suliman, Ahmed Alkhayyat, Ali Ihsan, Lihng LU

**Affiliations:** 1 Department of Electrical Engineering, Ferdowsi University of Mashhad, Mashhad, Iran; 2 Department of Optics Techniques, Al-Mustaqbal University, 51001 Hilla, Babylon Iraq; 3 Department of Pharmaceutics, College of Pharmacy, University of Al-Ameed, Karbala, Iraq; 4 Department of Computer Science, Al Rafidain University College, Bagdad, Iraq; 5 Department of Clinical Laboratory Sciences, College of Applied Medical Sciences, King Khalid University, Abha, Saudi Arabia; 6 College of Technical Engineering, The Islamic University, Najaf, Iraq; 7 Department of Medical Laboratories Techniques, Imam Ja’afar Al-Sadiq University, Al-Muthanna, 66002 Iraq; 8 School of Computer Science and Technology, Heyang Normal University, Heyang, Huan, 420012, China, Heyang, China

**Keywords:** Patient isolation, Tumor detection, Neural network, MRI image, Cancer, Engineering

## Abstract

MRI imaging primarily focuses on the soft tissues of the human body, typically performed prior to a patient's transfer to the surgical suite for a medical procedure. However, utilizing MRI images for tumor diagnosis is a time-consuming process. To address these challenges, a new method for automatic brain tumor diagnosis was developed, employing a combination of image segmentation, feature extraction, and classification techniques to isolate the specific region of interest in an MRI image corresponding to a brain tumor. The proposed method in this study comprises five distinct steps. Firstly, image pre-processing is conducted, utilizing various filters to enhance image quality. Subsequently, image thresholding is applied to facilitate segmentation. Following segmentation, feature extraction is performed, analyzing morphological and structural properties of the images. Then, feature selection is carried out using principal component analysis (PCA). Finally, classification is performed using an artificial neural network (ANN). In total, 74 unique features were extracted from each image, resulting in a dataset of 144 observations. Principal component analysis was employed to select the top 8 most effective features. Artificial Neural Networks (ANNs) leverage comprehensive data and selective knowledge. Consequently, the proposed approach was evaluated and compared with alternative methods, resulting in significant improvements in precision, accuracy, and F1 score. The proposed method demonstrated notable increases in accuracy, with improvements of 99.3%, 97.3%, and 98.5% in accuracy, Sensitivity and F1 score. These findings highlight the efficiency of this approach in accurately segmenting and classifying MRI images.

## Introduction

Cancer is the term used to describe the unregulated and aberrant growth of cells within the human body^1,2^. A brain tumor is distinguished by the formation of a mass and atypical cellular growth and division inside the brain tissue. According to^3–5^, the formation of a brain tumor occurs as a result of aberrant cellular development within the brain. Brain tumors exhibit malignancy due to their ability to displace vital brain tissues, thus impeding the performance of key bodily functions by occupying space within the brain. Due to their very aggressive nature, brain tumors exert a profound impact on the brain, which is often recognized as the most essential organ in the human body^6,7^. The optimal therapeutic approach for this particular ailment is contingent upon the timely identification of the tumor and an evaluation of its advancement^8–10^. The manual determination of tumor extent in brain images is currently utilized in clinical applications; however, due to its impracticality for processing enormous volumes of data, alternative methods are required^11,12^.

The segmentation of tumors or abnormal areas using magnetic resonance imaging (MRI) is of utmost importance in clinical and cancer research endeavors^6,13,14^. The accurate delineation of tumors by radiologists is commendable; yet, this process is time-consuming. Empirical evidence has demonstrated that utilizing approximation segmentations can be satisfactory for the purpose of indexing magnetic resonance imaging (MRI) databases. Brain tumors, albeit few, are classified as highly lethal malignancies. Gliomas, a distinct type of brain tumor, mostly consist of glial cells^15,16^. The aforementioned brain tumors represent the most commonly occurring type, which has garnered significant attention in contemporary research focused on the segmentation of brain tumors.

Early detection of brain cancers considerably enhances the probability of successful therapy and patient survival^17^. Brain tumors can be identified and characterized by the use of magnetic resonance imaging (MRI). This laboratory imaging technique uses radio waves to activate specific tissues, resulting in the generation of internal images^18,19^. These images are altered by a powerful magnetic field, allowing for the visualization of the tumor's shape, size, location, and metabolic activity. The acquisition of pictures in magnetic resonance imaging (MRI) is influenced by the excitation and repetition periods, which contribute to the formation of distinct MRI sequences. Various magnetic resonance imaging (MRI) techniques generate tissue contrast images of different sorts^20^. Consequently, they provide significant data for the purpose of tumor segmentation and diagnosis.

Image segmentation is employed to separate the sick region from the surrounding areas within the image. The use of a precise strategy for segmenting tumor size and location plays a crucial role in enhancing the efficacy of treatment planning. In order to initiate this process, it is necessary to have the initial parameters established by a competent professional or to have training data readily available^21^. Numerous investigations have been conducted to extract visual data from medical photographs with the aim of discerning different forms of tumors^22^. The expanding corpus of brain magnetic resonance (MR) images has provided neurosurgeons and medical researchers with more opportunities. However, the demand for meticulous data processing and diagnosis has become burdensome^23–25^. Hence, the utilization of computer-aided diagnosis can enhance the diagnostic capabilities of medical practitioners and reduce the duration required for accurate diagnoses.

Current clinical investigations commonly involve the analysis of magnetic resonance (MR) images, employing both primary quantitative criteria and exclusively qualitative criteria^26,27^. Hence, the enhancement of diagnosis and treatment planning necessitates the substitution of conventional assessments with image processing routines and tumor infrastructure measurements that are both extremely reliable and precise. These automated procedures may effectively examine brain tumor scans. The segmentation of glial tumors serves as the fundamental basis for a significant portion of the latest algorithms employed in the study of brain tumor targets^28–30^.

In order to safeguard the well-being of adjacent tissues, it is imperative to surgically excise the tumor prior to initiating any therapeutic interventions^31^. The process of segmenting a brain tumor involves the identification and differentiation of the tumor's tissues from the adjacent tissues. The manual execution of this approach is performed during routine therapeutic interventions^32^. In recent years, the research of automatic segmentation methods has gained prominence due to the time-consuming nature of manual segmentation, prompting the need for more efficient segmentation techniques. Segmentation is an essential undertaking in the field of medical imaging, which can be carried out through manual intervention by a proficient expert with a reasonably high level of precision. However, this process is time-consuming^33^. Nevertheless, the reliability of fully automated and accurate segmentation systems remains uncertain. Currently, there have been modifications made to partial automatic segmentation systems for their application in clinical settings^34^. The demanding and labor-intensive nature of the tasks performed by radiologists necessitates the development of semi-automated segmentation approaches^35^. Furthermore, by granting radiologists authority over the segmentation process, the limitations associated with the automatic segmentation method can be mitigated. Several semi-automated systems have been developed that require user initialization^36^.

Based on the review of the existing works, the gaps in this study can be examined as follows: (a) Low accuracy in the diagnosis and segmentation of tumors that many traditional methods and even some newer methods do not have sufficient accuracy in the diagnosis of tumors and need to be improved in this There is a context. (b) Limited use of features Some methods use a limited number of features that cannot include all the important information in MRI images, so the use of more complex and multidimensional features is necessary. (c) The complexity and time-consumingness of the diagnosis process, that the existing methods may be complicated and time-consuming, which can reduce the efficiency and speed of tumor diagnosis.

Accurate diagnosis of brain tumors from MRI images is one of the basic challenges in the field of medicine, which is of great importance from the point of view of early diagnosis and effective treatment of this type of cancer. However, conventional methods for this diagnosis are usually time-consuming and require human intervention. In this regard, providing automatic and accurate methods for diagnosing brain tumors from MRI images is considered a basic goal in medical research.

The main goal of this research was to provide an automatic and accurate method for detecting brain tumors from MRI images. Due to the time-consuming and high accuracy required in the diagnosis of this type of cancer, the use of automatic methods and data transfer from MRI images can help speed up and more accuracy in the diagnosis and treatment of brain tumors. By combining different algorithms such as Convolutional Neural Networks (CNN), Watershed, and Random Forest, this research achieves an innovative and accurate diagnostic method for brain tumors from MRI images.

One of the main innovations of this research is the use of a combination of different algorithms to detect brain tumors. In particular, the combination of Convolutional Neural Networks (CNN), Watershed, and Random Forest gives a lot of novelty and added value to the diagnostic method presented in this research. Also, this research shows that the use of markers in the Watershed algorithm and the application of a number of decision trees in the random forest lead to a significant improvement in the accuracy and efficiency of brain tumor detection from MRI images.

Overall, this research has added great value to the field of brain cancer diagnosis and treatment by providing an innovative and accurate diagnostic method for brain tumors from MRI images. The use of automatic and data-based methods in the diagnosis of brain tumors, in addition to increasing the speed of diagnosis, will also lead to greater accuracy and efficiency, and ultimately can help improve treatment results and the lives of patients. The subsequent section provides an overview of the authors' contributions to the present investigation.

- Combination of different algorithms for tumor detection: In this study, the combination of three powerful and widely used algorithms including Convolutional Neural Networks (CNN), Watershed Algorithm and Random Forest was used. This combination improves the accuracy and efficiency of the tumor detection method. Each of these algorithms has its own advantages and strengths, and by combining them, the proposed method benefits from all these advantages.
- Improving the Watershed Algorithm Using Markers: The Watershed Algorithm is typically sensitive to changes in image surface brightness, which can lead to additional segmentation. In this study, by using markers, this problem has been solved and segmentation accuracy has been improved. By eliminating false regional minima, markers reduce the problem of sensitivity to illumination changes and prevent false segmentation.
- Using a large number of decision trees in the random forest: Using a large number of decision trees in the random forest algorithm has increased the accuracy of tumor detection. Using random feature selection techniques, this method reduces the instability of individual decision trees and leads to overall improvement of model performance.

The rest of the article is organized as follows. In the second part, the background of tumor detection accuracy in medical images is given. The proposed approach is discussed in detail in the third section. In the fourth section, the evaluation and results are stated. Finally, the conclusion is given in the fifth section.

## Background

Brain tumor analysis represents a critical area in medical imaging necessitating precise and efficient techniques for early detection and diagnosis. Magnetic resonance imaging (MRI) stands as a prevalent diagnostic method for identifying brain tumors. Nonetheless, achieving accurate and reliable analysis of MRI images for brain tumor diagnosis poses a formidable challenge, even for seasoned radiologists. Deep learning (DL) has emerged as a promising solution, demonstrating significant success in various medical image analysis tasks, including segmentation, classification, and tumor detection^37^. In the proposed method, MRI images undergo pre-processing using a Gaussian wavelet filter. Subsequently, a pre-trained AlexNet model is employed for feature extraction. These extracted features are then utilized to train a hybrid CNN-LSTM deep learning model, which classifies the images into four distinct classes^38^.

The latest advancements in brain tumor detection systems (BTDS) are showcased to provide inspiration for upcoming researchers to devise novel architectures for accurate and efficient tumor detection. In this context, multimodal brain tumor segmentation data is utilized, undergoing registration, skull cropping, and high contrast iron volume histogram matching.

In a study by^39^, a capsule network (CapsNet) is tailored specifically for brain tumor classification. The outcomes of cutting-edge deep neural network (NN) architectures for tumor detection are analyzed and elucidated. Among these architectures, VGG16 and CapsNet demonstrate the highest f1 score and precision values, with VGG19 following closely behind. created an effective and automated method for diagnosing AD using MRI image data that is based on deep learning^40^. Data collecting is the first stage in AD classification; specifically, it is concerned with gathering MRI scans of the brain from the ADNI and the OASIS datasets. An improved median filter (IMF) is used for preprocessing the obtained data, the [0, 1] rescaling approach is employed for normalisation, and morphological thresholding is employed for skull segmentation. The Multiview Fuzzy Clustering (MvFC) algorithm is used to partition the brain tissues into grey matter (GM), cerebrospinal fluid (CSF), and white matter (WM) using the preprocessed images^40^. Magnetic resonance imaging (MRI) is widely utilized for diagnosing neurodegenerative disorders owing to its exceptional spatial resolution. In a study referenced as^41^, the authors employed a bidirectional short-term memory with deep attention (DABiLSTM) utilizing stochastic gradient optimization (SGDO) for Alzheimer's Disease (AD) diagnosis. A bidirectional Gaussian filter is applied for preprocessing brain MRI images. Subsequently, the abnormal regions of the image, identified using BiLSTM, are segmented utilizing depth attention.

Examining magnetic resonance imaging (MRI) images is crucial for tumor analysis in patients. However, due to the substantial volume of generated data, manual techniques often result in numerous misclassifications. To mitigate misclassifications and manage the large dataset effectively, the proposed work introduces a deep learning-based classification model comprising five primary modules. Initially, the images undergo cropping and filtering via a cranial guided bidirectional filter (GBF). Subsequently, tumor regions are segmented utilizing a thresholding scheme, followed by the extraction of main tissue and edge features using the improved Gabor wavelet transform (IGWT)^42^.

In^43^, examined the application of intensity normalization as a preliminary procedure. Additionally, they employed data augmentation in conjunction with an automatic segmentation approach, a practice that is not commonly observed in convolutional neural network (CNN)-based segmentation methodologies. The objective of their study was to segment tumor images. Brain magnetic resonance imaging (MRI) scans have proven to be highly valuable in clinical and research settings. In addition, Liu et al.^44^ introduced a novel convolutional neural network (CNN) structure that effectively leverages both local information and broader global context features. The subsequent convolutional neural network (CNN) in a cascade design leverages the output of a first CNN as an additional source of information. A proficient wavelet-derived texture feature framework for classifying brain MRI images from two distinct modalities is delineated in^45^. Features are extracted from MR images utilizing this proposed model. Subsequently, principal component analysis (PCA) is employed to reduce feature dimensions. The resultant features are then utilized to train various classifiers, with validation conducted via tenfold cross-validation.

In^46^ developed a deep learning model that utilizes a back-propagation neural network classifier for the purpose of predicting stroke based on CT/MRI scan data. The efficacy and accuracy of the proposed model are evaluated and compared to those of the existing models, resulting in findings characterized by a notable level of sensitivity, specificity, accuracy, and precision. In^47^, introduced a convolutional neural network (CNN) model with the aim of accurately identifying different classifications of brain tumors. The suggested network structure exhibited exceptional performance, with an impressive total accuracy of 96.05%. The findings provided empirical evidence for the hypothesis that effective preprocessing techniques and data augmentation strategies contribute to precise classification outcomes. Moreover, the results showcased the capability of the proposed model to successfully categorize brain tumors across diverse applications. The efficacy of different approaches in locating pre-existing entities has been evaluated. Zhang et al.^48^ employed magnetic resonance imaging (MRI) to accurately identify the precise locations of distinct brain tumors. Their primary objective was to develop a robust and efficient system utilizing the transfer learning approach. In this study, pre-trained models like Xception, ResNet18, VGG19, and ResNetV2 were employed to extract deep features from brain MRI data. Based on empirical evidence, their proposed Convolutional Neural Network (CNN) model demonstrates superior performance compared to the other three models that were put forward.

According to the review of the works done in this section, some of the disadvantages and gaps of the past works can be summarized as follows:

- Low accuracy in diagnosis: traditional methods such as using computational features or simpler algorithms may have low accuracy in diagnosing and classifying tumors. This is usually due to their inability to examine more complex features and different variables in MRI images.
- Use of limited features: some of the past methods may not be able to consider all dimensions and changes in MR images due to the use of limited or inappropriate features. This can lead to the loss of important information and reduce the accuracy of tumor diagnosis.
- Complexity of the diagnosis process: existing processes for the diagnosis of tumors may be complex and time-consuming. This can reduce the efficiency and speed of tumor detection and prevent practical use in clinical and medical environments.

The purpose of preliminary Table 1 is to compare and evaluate the efficiency of different methods in the diagnosis and classification of brain tumors from MRI images that have been used in previous studies. This table presents the Dice Similarity Coefficient results for these methods and is used as a measure to evaluate the accuracy and efficiency of these methods. Table 1 shows the Dice similarity percentage for several methods of brain tumor detection from MRI images that have been investigated in different years and using different BraTS datasets. Based on the data presented in the table, the Dice similarity results range from 79.32% to 92.46%. These results show that different methods have different accuracy in the diagnosis and classification of brain tumors. The obtained results show that newer methods such as capsular neural networks (CapsNet) and convolutional neural networks (CNN) can significantly improve the accuracy of diagnosis and classification of brain tumors. have a brain from MRI images. This table shows well that the use of advanced and combined techniques can lead to an increase in the accuracy and efficiency of brain tumor detection methods and serve as a basis for the development of new and improved methods in this field.

**Table 1:** Efficiency results of some previous studies.

References provided	Data set	Whole-scale dice similarity coefficient (%)
^37^	BraTS 2017	80.10
^38^	BraTS 2012	91.24
^40^	BraTS 2018	79.32
^41^	BraTS 2018	88.20
^43^	BraTS 2012	83.14
^44^	BraTS 2017	86.59
^45^	BraTS 2014	92.02
^46^	BraTS 2018	85.36
^47^	BraTS 2016	92.46
^48^	BraTS 2012	90.84

## Proposed method

**Figure 1:**
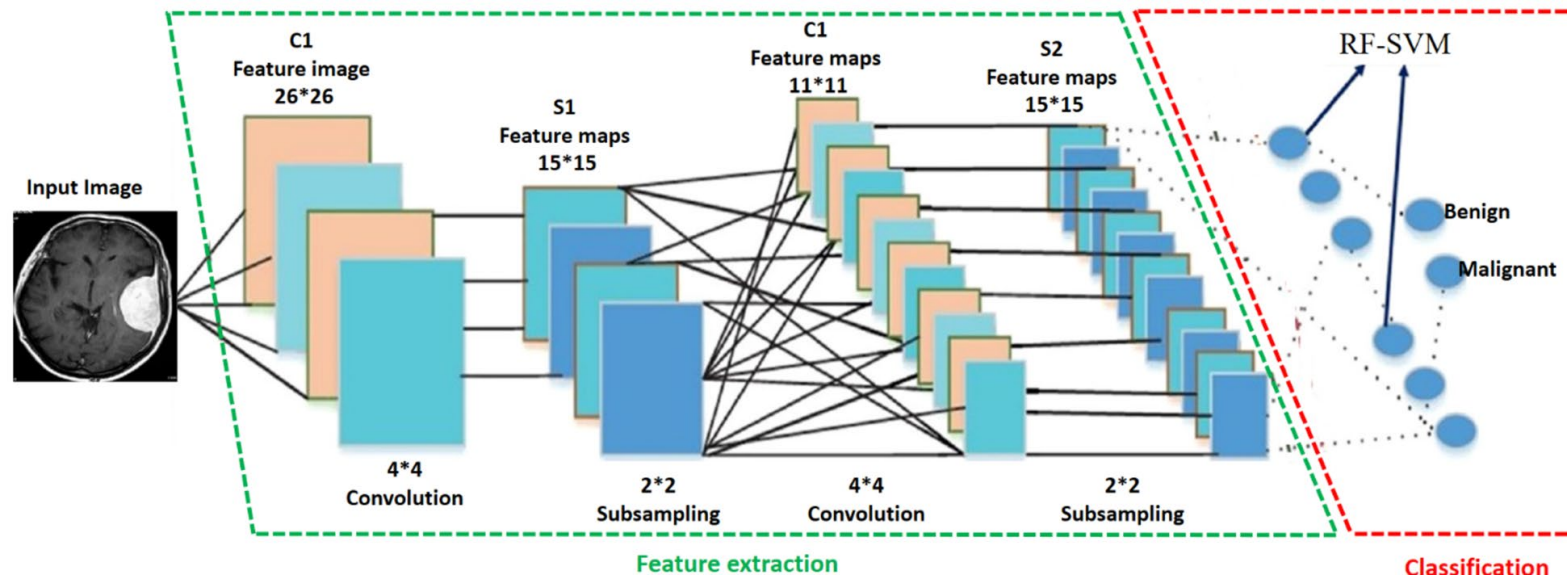
The general structure of the proposed solution.

In this study, we introduce a hybrid approach termed HCNN-RF to enhance the effectiveness of tumor identification. To eliminate noise from the images, preprocessing operations are conducted initially. The image output from the preprocessing stage is then utilized, and segmentation is performed using the Watershed method. The accuracy of feature evaluation by the feature extraction department will be significantly influenced by the segmentation result. Convolutional neural networks (CNN) are employed to extract key properties from segmented images. Feature extraction aims to reduce the original dataset by measuring specific features^49^. The extracted features serve as input for the categorization section. Finally, tumors are identified using classification based on the random forest (RF) method. RF is one of the most potent maximum vote classification methods, enabling accurate tumor diagnosis when applied to the collected data and learned from it. The various processes constituting the solution described in this article are depicted in Fig. 1.

### Pre-processing

The preprocessing stage improves the quality of input photos for segmentation. Additionally, preprocessing aids in enhancing certain aspects of the images, such as maintaining and smoothing the edges, eliminating unnecessary noise, enhancing the signal-to-noise ratio, and removing unwanted background elements. Therefore, in this approach, preprocessing procedures are carried out using grayscale techniques and a morphological filter. To accomplish this, the image is first converted to black and white using the grayscale filter, and then noise is removed from the image using the morphological filter. In this method, the final value of the pixel is determined by the average value of the pixels within the kernel. According to evaluations^43^, the morphological filter offers superior end quality compared to other noise removal techniques.

### Segmentation of water spreader

The most crucial phase of image analysis, known as image segmentation, is where the information contained within the image (such as its edges, perspectives, and individual region identities) is extracted. Based on this, one of the most effective and widely used image segmentation techniques applied in this study is the segmentation of MRI images using water diffusers^49,50^. This approach offers the benefits of being straightforward, quick, and accurate. In this method, the picture gradient serves as the area for image processing. Typically, uniform tissues exhibit low gradient values in an MRI image; thus, these points are identified as valleys, while the image borders are identified as peaks.

**Figure 2:**
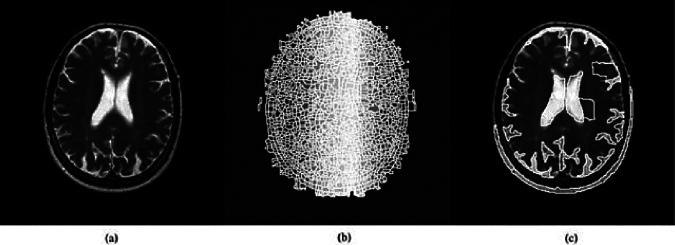
The effect of markers on segmentation using the Watershed algorithm. (**a**) Primary image; (**b**) the result of applying the Watershed algorithm without markers; (**c**) The result of applying the Watershed algorithm through the use of markers.

For magnetic resonance image segmentation, the water diffuser approach combines the two principles of edge detection and mathematical morphology (pixels with a consistent brightness gradient). However, the fundamental flaw of the water diffuser solution lies in its sensitivity to brightness changes in the image surface, which leads to additional areas in the image and can reduce segmentation accuracy. Therefore, in this study, markers are utilized to address this issue. Markers eliminate spurious regional minima through interactive zoning, thus resolving the problem of sensitivity to variations in picture brightness. Subsequently, the gradient of a new image is obtained, mitigating the issue of over-segmentation and minimizing the occurrence of incorrect regional minima^51,52^. Figure 2 illustrates the effect of markers on enhancing efficiency in the Watershed algorithm.

### Feature extraction

**Figure 3:**
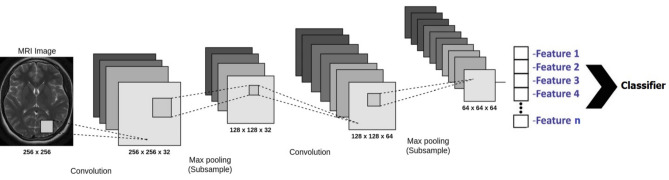
How to extract features using CNN.

The desired features in an image are found and used for additional processing using the feature extraction method. By assessing specific attributes, picture extraction aims to minimize the original data set. The classifier considers the retrieved features as input. In this article on convolutional neural networks, many methods are utilized to extract the feature. One of the best and most effective configurations suggested for use in modeling neural networks, which consist of an input layer, one or more hidden layers, and an output layer, are these networks. In the suggested solution, Fig. 3 illustrates how to leverage these networks for tumor identification. As can be seen, the primary features of the MRI picture are retrieved in the first layer, known as the convolution layer, and are then pooled in the subsequent layer. Only the primary features should remain in this layer after the dimensions of the features are shrunk. The classifier in this study is based on random clustering, and it receives the output of this layer.

Each layer in this network contains a unique weight matrix, bias vector, input vector, and output vector. The number of neurons in different layers can vary. An output layer is a layer whose output is the network's output. Hidden layers are the other layers. Networks with multiple layers are more effective than those with only one layer. An example of a two-layer network that can be trained to approximate most optional functions is one that has a first sigmoid layer and a second linear layer. Two or three levels are typical for application networks. Since we calculate an F0 value for each signal, we will have one output neuron. The number of neurons in the input layer is equal to the number of features of the input vector, which in this case is equal to the number of algorithms used to extract F0. For each signal, F0 is determined in this situation. The neural network receives these values as input. The synaptic weights are multiplied by the input values before being transferred to the hidden layer. In the first order, the weights are chosen at random, but it's crucial to choose the number of hidden layers and neurons in each one because if they're few, the network won't have enough other resources to deal with challenging nonlinear situations. There will be two issues if it is too much. First, the network's training period lengthens; second, the network might pick up trivial training data and struggle to solve problems^53,54^. Typically, trial and error is used to determine the hidden layer's neuron count. In this problem, a hidden layer of 20 neurons has been taken into consideration based on the best experimental findings for the intended network. The following formula^55^ is used to determine the output for the hidden layer:

1 $$OP=SGM (\sum {x}_{i} {w}_{tm}^{h})$$

Once more, by doubling the weight vector, the output of the hidden layer is passed to the output layer^56^. It can be demonstrated as follows based on the appearance of the i_th_ nerve output (in the bottom layer):

2 $$Oi=Sgm ({\sum }_{m}Sgm (\sum {x}_{i} {w}_{tm}^{h}){w}_{mi}^{o})$$

where *h* and *O* represent the hidden layer and the output layer, respectively, and w means the weights of the layers. Sgm is also a sigmoid function which is defined as follows^57^:

3 $$Sgm= \frac{1}{1+ {e}^{-x}}$$

In neural networks, learning essentially entails changing the synaptic weights to align the input–output relationship of the neuron with a predetermined objective. The training of neural networks has been approached in a variety of ways. The algorithm for error back propagation is one of these techniques. In order to determine the network error, the network output is compared to the ideal output. To reduce this inaccuracy, the network's output is calculated at each step, and the weights are adjusted in accordance with how it differs from the expected output. The square of the error between the outputs of the network and the goal function is attempted to be minimized in this method by employing the slope gradient due to its simplicity and widespread use. In reality, the error backpropagation approach, which is the method utilized in 95% of contemporary neural network applications, is the most popular method to reduce the error. After computing the prediction error, this approach gradually moves the synaptic weights from the top layer to the bottom layer to reduce the prediction error. In actuality, this method is used to find errors on the nodes of the previous and subsequent layers. The error is described in this instance as follows^58^:

4 $$E(\overrightarrow{w})=\frac{1}{2} \sum_{d\in D}\sum_{k\in outputs}({t}_{kd}-{o}_{kd})2$$

“Outputs" refers to the target value, the output corresponding to the kth output unit, training example d, and the set of units from the output layer, t_kd_, and o_kd_. It should be emphasized that the slope gradient approach seeks to minimize error in order to arrive at an appropriate hypothesis^59^. However, there is no assurance that this algorithm will arrive at the minimal value. After the error propagation, two categories of weights should be changed in each step of the algorithm's execution, presuming the hidden layer exists. the weights of the connections from the input layer to the hidden layer and from the hidden layer to the output layer, respectively. The error is determined as follows for the output layer^60,61^:

5 $${\delta }_{k}= {O}_{k}(1-{O}_{k})({t}_{k}-{O}_{k})$$

The error is identified as follows for the buried layer:

6 $${\delta }_{k}= {O}_{k}(1-{O}_{k}){\sum }_{k}{w}_{kh}{\delta }_{k}$$

Lastly, the distribution of each weight is changed as follows:

7 $${w}_{ji}= {w}_{ji}+ {\Delta w}_{ji}$$

which in the above relation:

8 $${\Delta w}_{ji}= \eta {\delta }_{j}+ {X}_{ji}$$

In this instance, these weights are adjusted repeatedly to achieve the ideal weights for the solution. The neural network is fed test data that it has never seen before in the subsequent stage, known as the test stage. Based on the weights acquired throughout the training phase, its *F*_*0*_ is determined. The network receives the sample information one by one at the start of the learning phase. The input data flows through the network, meaning that it is multiplied by the synaptic weights, and the output of each neuron's activity, which takes the form of a signal, serves as the input for the neurons in the layer below. Each sample's information flow will finally come to an end with a response from the network at the output layer. The calculated response will differ from the experimentally measured stability because random synaptic weights are used. For the network to provide a good forecast, this difference that is, the difference between the amount of response anticipated by the network and its experimentally measured quantity should be near to zero. Following that, the second sample's data enters the network. Naturally, the new sample will have an error once more if the synaptic weights remain the same. In order to reduce error (both in this example and the instances before it), the process of post-propagation of the error is once more applied, and the weights are adjusted accordingly. This allows the network to assess the full n-dimensional space of parameter relationships once there are enough examples entered into it. The network is said to be covered in this instance. It indicates that the most concave point on the prediction error curve has been reached. This indicates that the learning process went well and that the overlay network is now prepared for prediction.

### Feature training

- Convolution layer construction:
  In several of our studies, we find that using five convolutional layers in our technique can yield good accuracy without requiring a lengthy training period. The following formula is used to change the data in the convolution layers using the ReLU nonlinear transformation function:
  9 $$f\left(x\right)= \left\{\begin{array}{ll}o & \quad if \; x < 0\\ x & \quad if \; x \ge 0\end{array}\right.$$
- Performance of the proposed method to determine the classification and duration of bleeding.
  The brain image was labeled with four different forms of hemorrhages, as previously mentioned: intracerebral, subdural, subarachnoid, and epidural. Doctors must, however, be aware of additional crucial details, such as the location and timing of the bleeding, in order to provide prompt treatment. As a result, we plan to compute the numbers provided in this section for the location and length of the cerebral hemorrhage, as detailed in Sections II, D, and E.

### Classification

The final step of the suggested strategy involves performing the causes associated with tumor classification. Using the retrieved features produced from CNN neural networks, tumor determination operations are carried out during this phase. This information is utilized to inform the classification algorithm based on random forest^62^. One of the numerous classification techniques is random forest. This approach creates classifiers either by rearranging the feature space vector or the training data. Random movement is used to move training data or feature vectors. The primary distinction between the Bagging method and the Random Forest method is the random feature selection. Every time a branch selection step is performed, RF first chooses a set of features at random, after which it continues the branch selection procedure inside of the feature set. When used as a group (voting) method, RF creates a variety of decision trees as basis classifiers and uses the results of the main trees in combination with the results of the majority vote. As a result, the random forest approach, which employs many decision trees, is produced. Each tree in this solution represents a discrete category. A sizable collection of these trained trees combined with data that was chosen at random results in a broad choice of the appropriate category. When compared to a single classification tree, the random forest algorithm can improve prediction accuracy. Small changes in the training set can cause instability in the individual tree, which disrupts the prediction accuracy in Although it is a test sample, the random forest algorithm's group structure enables it to adjust to changes and get rid of instability. The following two components make up the two primary random processes that form the foundation of this solution.

- The same bagging procedure 3 is used for bootstrap samples when performing random sampling of data.
- Choosing input qualities at random to create single decision trees.

Only a small portion of each node is used for random selection, enabling the search for the ideal partition. Since both the training set and the bootstrap are N-dimensional, a sample of the learning set—roughly two-thirds of it—is included in the bootstrap set. The bootstrap set discards one-third of the learning samples^63^. These samples are the actual OOB collection. Each tree is thereafter created in its own bootstrap set and evaluated against the OOB set. The average categorization error in OOB sets pertaining to every tree in the forest is equal to the overall OOB error. In this instance, relation 4 establishes the upper bound of the error:

10 $$GE= \rho (\frac{1}{{S}^{2}}-1)$$

**Figure 4:**
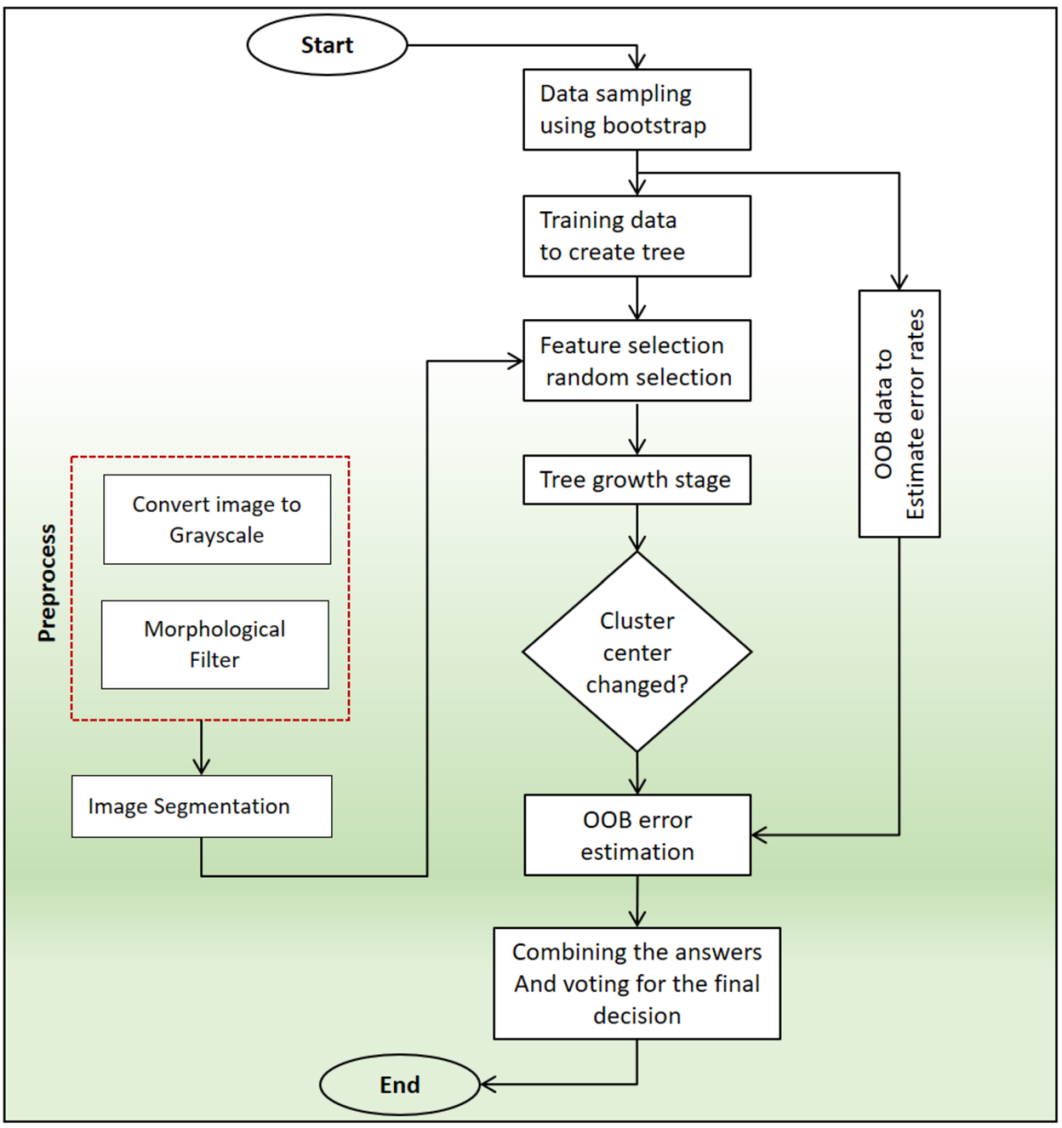
Decision-making flowchart for the random forest method.

In the connection shown above, $\rho$ stands for the average value of reliance, and *S* also denotes the category's strength. As a result, the correlation should be decreased and the power factor should be raised in order to decrease the inaccuracy. In other words, the smaller the ratio, the better the forest performs and the smaller the inaccuracy. This ratio is calculated by dividing dependence by the square of power. Each tree generates a judgment independently for classification reasons; the choice is then determined as follows once all decisions are gathered and combined:

- Every tree has an equal vote, and decisions are made by majority vote.
- a weighted sum of trees where the learning mistake serves as the basis for the weighting.
- In this simple Bayes combination, the weights are taken into account based on the prior probabilities of the classifications. The flowchart of random forest algorithm is shown in Fig. 4.

In the paper, several methods and techniques are employed to mitigate overfitting and under-fitting and ensure a balance between model complexity and generalization performance. One prominent approach involves the utilization of a regularization technique known as L2 regularization, also referred to as weight decay. This technique imposes a penalty on the squared magnitude of the model's parameters, effectively discouraging overly complex models by penalizing large parameter values. By incorporating L2 regularization into the model architecture, the study aims to prevent overfitting by encouraging the model to generalize better to unseen data while still capturing relevant patterns in the training data.

Furthermore, the chosen model architecture employs dropout regularization, a technique commonly used in neural networks to mitigate overfitting. Dropout randomly deactivates a fraction of neurons during each training iteration, forcing the model to learn redundant representations and preventing it from relying too heavily on any individual neuron. This helps to promote model robustness and generalization by ensuring that the network does not become overly specialized to the training data. Regarding the implications of overfitting and under-fitting on interpretability and robustness, it is essential to note that an overfitted model may produce overly complex decision boundaries that are difficult to interpret and prone to capturing noise in the data. Conversely, an under-fitted model may oversimplify the underlying relationships in the data, leading to poor generalization and limited predictive performance on unseen instances. By effectively addressing over- and under-fitting issues, the study aims to enhance the interpretability and robustness of its results, ensuring that the model captures meaningful patterns in the data while avoiding spurious correlations or overly simplistic representations. This not only enhances the reliability of the research findings but also facilitates their applicability in real-world scenarios, where robust and interpretable models are essential for informed decision-making.

The proposed method named HCNN-RF is a hybrid approach for tumor detection in MRI images that uses the combination of Convolutional Neural Networks (CNN) and Random Classification Algorithm (RF). This innovative method by using MRI images, different filters to improve the quality of images, segmentation method based on Watershed algorithm, extraction of required features and finally using convolutional neural network and RF classification algorithm, has been able to with high accuracy and significant difference Diagnose brain tumors with existing methods.

On the other hand, the HCNN-RF method pays attention to features such as the combination of two strong algorithms (CNN and RF), the use of advanced methods such as segmentation using the Watershed algorithm, and the extraction of salient features from MRI images, which results in high flexibility and accuracy. brings. Moreover, using a diverse dataset, it shows significant improvements in accuracy, sensitivity, and F1 score over existing methods. This method not only uses previous ideas, but by combining and optimizing these ideas, it provides an innovative and efficient method for tumor detection in MRI images.

Algorithm 1 is designed to detect brain tumor from MRI images using HCNN-RF approach, a combination of convolutional neural networks (CNN) and random forest (RF) algorithm. In order to improve the accuracy of tumor detection from magnetic resonance images, this combined method consists of the following steps:

- Preprocessing: In this step, MRI images are converted to grayscale and morphological filters are used to remove noise. This process includes converting to black and white images, removing noise using morphological filters, and improving the edges and smoothing the images, and finally the images are ready for the next step, Segmentation.
- Segmentation using the Watershed method: In this step, using the Watershed transformation and markers to guide the segmentation, the areas of the images that are related to the tumor are extracted with high accuracy. This process is based on the image gradient, and by using markers, it is used to increase the accuracy and reduce the error in segmentation.
- Feature extraction using CNN: By completing the segmentation step, important features are extracted from the images. This feature extraction is done using Convolutional Neural Networks (CNN) which analyzes the structural and morphological features of the images. These features are used as input for the next part, Classification.
- Classification using RF: In the final step, the random forest (RF) algorithm is used for tumor classification and diagnosis. As a powerful classification method based on decision making among several decision trees, RF offers high accuracy and power in tumor detection from MRI images.

This algorithm has achieved a significant improvement in the accuracy and efficiency of tumor detection from MRI images by using different steps and complex methods. With the successful combination of CNN and RF and the use of appropriate optimization and evaluation methods, this is a suitable method for future research in the field of brain tumor detection from MRI.

**Algorithm 1:**
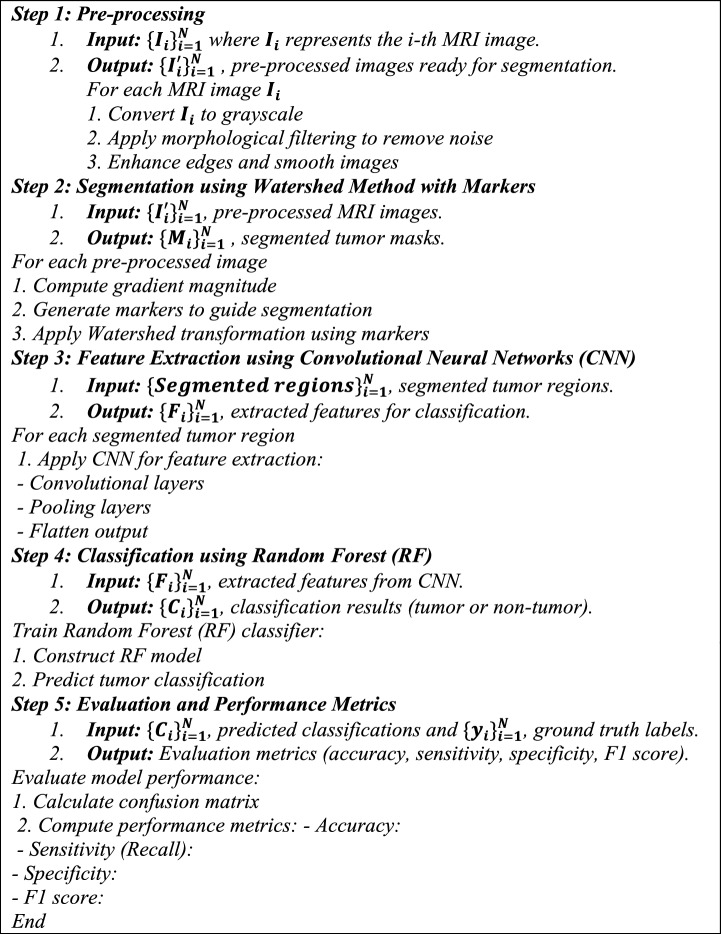
Brain tumor detection from MRI images (HCNN-RF approach).

## Discussion and evaluation

Computer-assisted brain tumor diagnosis has attracted the attention of numerous researchers as a result of the advancement of computational intelligence and machine learning techniques, making it a crucial diagnostic tool in diagnostic radiology and medical imaging. The previous section introduced a suggested method for threshold-based segmentation based on the cooperative HCNN structure and RF methodology. This made it possible to create a system for automatically detecting brain tumors and providing effective treatment. In this section, the initial set of data used and the performance evaluation standards are presented, followed by the outcomes of the suggested approach and the comparative analysis. It should be mentioned that the proposed strategy was assessed using k-fold cross-validation (CV). This method of performance estimation is typical.

The original sample is randomly split into k equal subsamples in K-fold CV. The remaining (k − 1) sub-samples are utilized as training data, while one sub-sample is preserved as validation data for model testing. Each of the k subsamples is then used exactly once as validation data once k iterations of the CV procedure have been completed. Then, an estimate can be created by averaging the k that results from the folds. Software from MATLAB 2022a is also used for implementation.

The method has been carried out using the BRATS 2019 dataset to assess the suggested solution. Since 2012, the size of the BRATS database of brain tumor magnetic scans has been growing. 16 files of the LG type and 158 files of the HG type were included in the dataset that was received. It was chosen to use only the HG data set because there aren't much data connected to LG. The HG dataset included a variety of categories from which 80 numbers were chosen to model 80 photos. For testing, 40 photos have been taken into account.

By calculating image similarity metrics like the Dice Similarity Score (DSS) and Jaccard Similarity Index (JSI) with the aid of a comparative analysis with ground truth (GT) of the image produced by the Sefexa image segmentation tool, the performance of the suggested segmentation method is assessed. The following criteria are expressed mathematically:

11 $$DSS=\frac{2({I}_{gt}\cap {I}_{s})}{\left|{I}_{gt}\right|\cup \left|{I}_{s}\right|}$$

12 $$JSI=\frac{({I}_{gt}\cap {I}_{s})}{\left|{I}_{gt}\right|\cup \left|{I}_{s}\right|}$$

where IS stands for the segmented image and I_GT_ for the ground truth image. The HCNN-RF model has been used to apply the suggested segmentation strategy for more in-depth studies. In Table 2, the segmentation methods and BMM-LA GMM-LA values on 5 brain tumor MR images are shown together with the DSS and JSI values for the suggested segmentation approach. The results in Table 2 demonstrate that the suggested segmentation method outperforms the GMM-LA and BMM-LA segmentation methods in terms of performance.

**Table 2:** Results of segmentation methods.

Segmention method	Measure	Image 1	Image 2	Image 3	Image 4	Image 5	Average
HCNN-RF	DSS	0.98	0.95	0.97	0.86	0.92	0.94
	JSI	0.96	0.93	0.91	0.80	0.89	0.89
BMM-LA	DSS	0.96	0.92	0.95	0.82	0.90	0.91
	JSI	0.89	0.88	0.90	0.75	0.82	0.84
GMM-LA	DSS	0.94	0.90	0.93	0.80	0.88	0.89
	JSI	0.85	0.68	0.82	0.72	0.78	0.77

**Figure 5:**
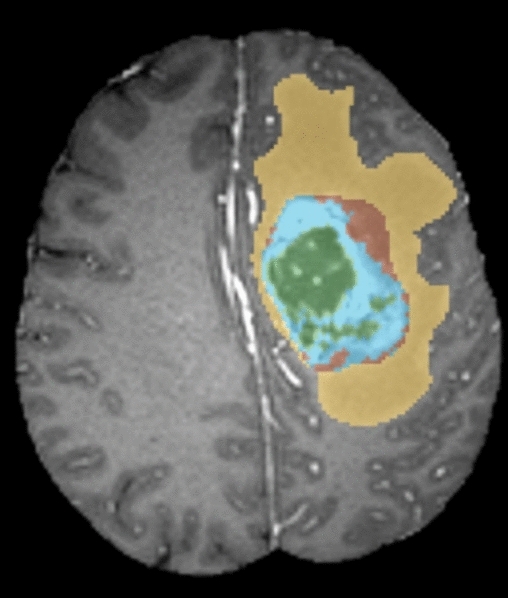
Typical tumor-related MR image.

**Figure 6:**
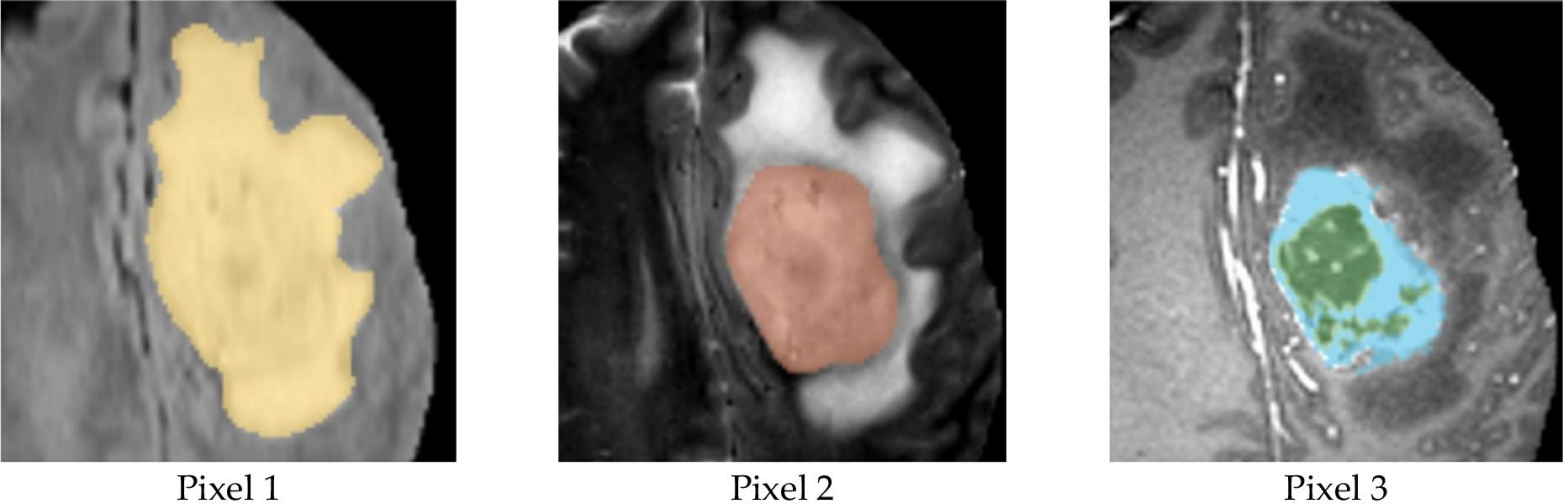
Three image combined into one after applying the suggested segmentation technique.

**Figure 7:**
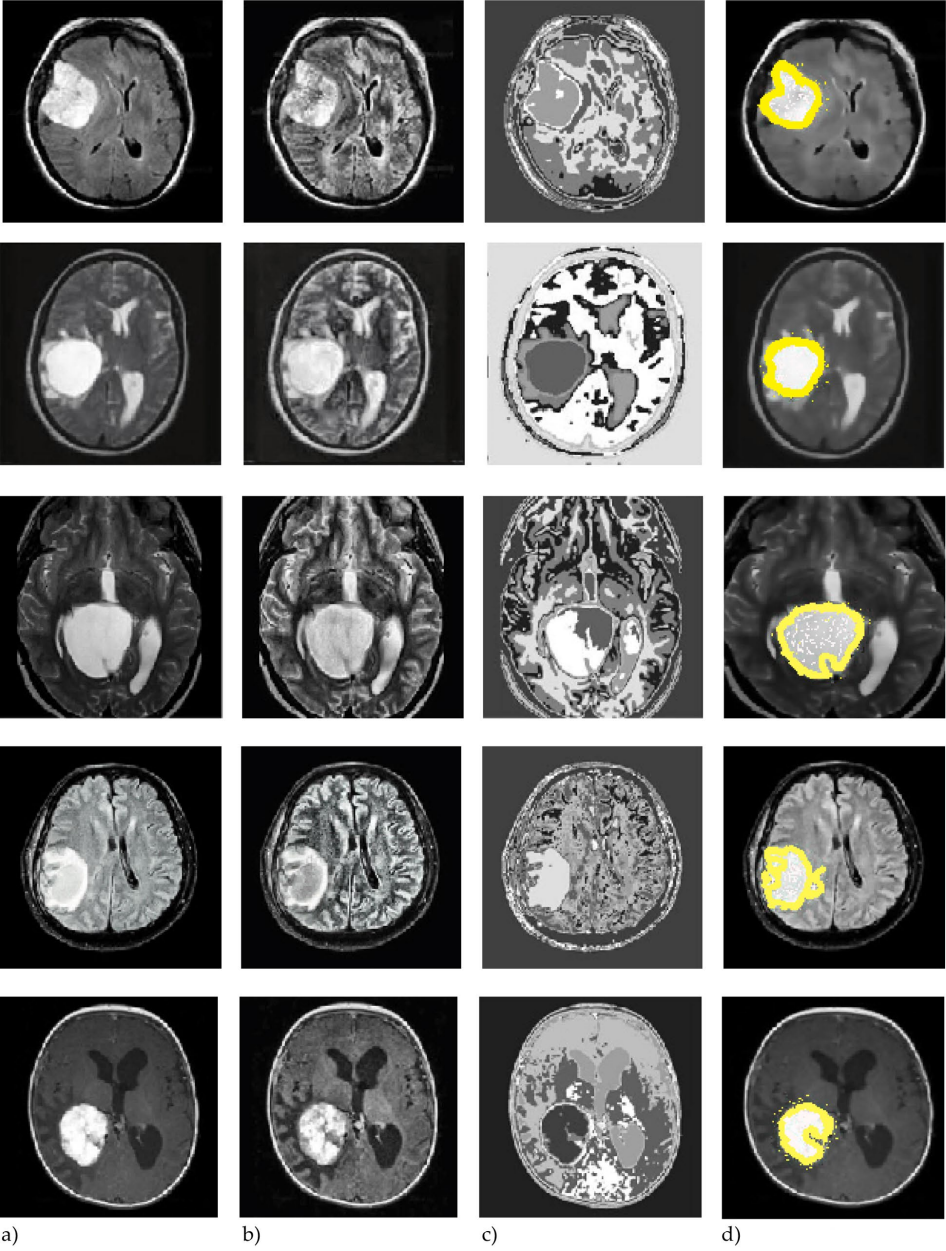
Accurate tumor extraction process from MRI images. (**a**) Tumor-containing primary MR images. (**b**) Enhanced pictures. (**c**) Fragments of tumor. (**d**) Segmented images.

**Figure 8:**
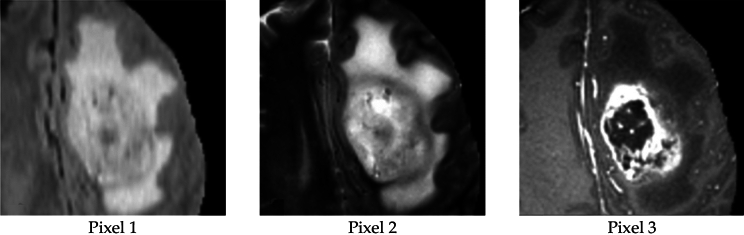
Image produced using the HCNN-RF approach after segmenting three images.

Figure 5 displays an example of a typical MR image. The three segments produced by applying the suggested segmentation method to the MR image displayed in Fig. 5 are presented in Fig. 6. Figure 7 shows that the suggested method successfully extracts the tumor from the original MR images. Figure 5 in Fig. 8 displays the outcomes of picture segmentation using the HCNN-RF segmentation technique. As shown in Fig. 8, three images are generated using the HCNN-RF approach after segmentation. The specificity, sensitivity, and accuracy of the suggested brain tumor detection system are three fundamental assessment criteria that are used to assess its performance.

13 $$Accuracy= \frac{TP+TN}{TP+TN+FP+FN+TP}$$

14 $$\text{Sensitivity}= \frac{TP}{FN+TP}$$

15 $$\text{Specificity}= \frac{TN}{TN+FP}$$

16 $$f1 Score=\frac{TP}{TP+\frac{1}{2}(FP+FN)}$$

where, in order, TN, TP, FN, FP, and FN indicate, respectively, true negative, true positive, false negative, and false positive. Tables 3 and 4 illustrate, respectively, the evaluation results of the brain tumor identification system without and with the segmentation process utilizing SVM (with different kernels), DT, and K-NN classifiers.

**Table 3:** Results of the system for diagnosing brain tumors without segmentation.

Classifier	Measures	Threefold (%)	Fivefold (%)	Ninefold (%)
SVM (kernel linear)	Accuracy	95.79	95.8	95.8
	Specificity	95.54	95.4	95.5
	Sensitivity	96.01	100	96.18
	F1 score	97.2	95.6	98.1
SVM (kernel polynomial)	Accuracy	92	93.26	93.27
	Specificity	92.61	95.58	92.96
	Sensitivity	91.49	91.22	93.63
	F1 score	86.6	90.6	94.2
SVM (kernel RBF)	Accuracy	65.42	69.2	70.48
	Specificity	25.24	33.18	36.17
	Sensitivity	100.86	99.8	99.5
	F1 score	84.9	100	99.6
Decision tree	Accuracy	93.23	92.02	95.77
	Specificity	92.93	95.5	95.54
	Sensitivity	93.27	89.05	95.96
	F1 score	95.1	89.2	90.2
*K*-NN (*K* = 1)	Accuracy	94.69	94.36	94.52
	Specificity	98.9	100	95.46
	Sensitivity	89.13	94.26	93.65
	F1 score	95.8	99.2	100
*K*-NN (*K* = 4)	Accuracy	93.8	94.4	94.54
	Specificity	85.22	93.55	93.09
	Sensitivity	99.4	95.21	96.03
	F1 score	93.6	95.8	100

**Table 4:** Results of the segmented evaluation of the brain tumor diagnosis system.

Classifier	Measures	threefold (%)	fivefold (%)	ninefold (%)
SVM (kernel linear)	Accuracy	97.07	98.34	99.6
	Specificity	97.98	98.26	98.29
	Sensitivity	96.39	98.57	95.8
	F1 score	82.5	86.7	100
SVM (kernel polynomial)	Accuracy	94.61	97.08	97.04
	Specificity	98.32	98.1	98.45
	Sensitivity	91.92	96.4	95.59
	F1 score	94.2	91.7	88.9
SVM (kernel RBF)	Accuracy	89.54	89.53	90.74
	Specificity	80.22	82.11	84.57
	Sensitivity	98.33	96.53	96.48
	F1 score	86.5	99.4	92.8
Decision tree	Accuracy	94.54	94.52	95.81
	Specificity	92.9	94.82	92.57
	Sensitivity	96.09	94.12	98.74
	F1 score	92.6	95.1	89.4
*K*-NN (*K* = 1)	Accuracy	100	100	99.57
	Specificity	95.02	97.4	98.20
	Sensitivity	91.47	94.47	98.23
	F1 score	91.8	90.5	92.5
*K*-NN (*K* = 4)	Accuracy	94.8	97.46	100
	Specificity	94.2	99.2	98.2
	Sensitivity	89.16	94.4	93.96
	F1 score	83.7	86.3	89.2

**Figure 9:**
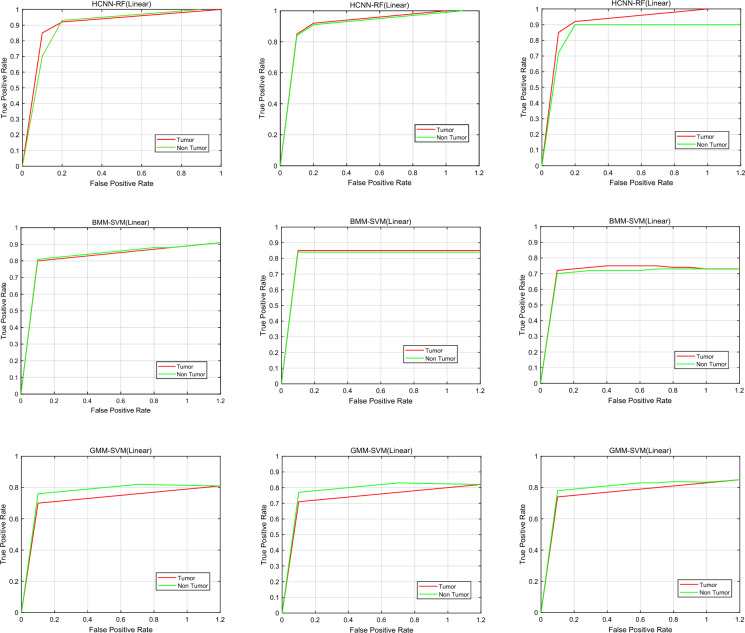
ROC chart comparing the accuracy of the HCNN-RF segmentation method for detecting brain tumors to that of the BMM-LA and GMM-LA. (**a**) Threefold, (**b**) Fivefold and (**c**) Ninefold.

The accuracy is higher when the SVM classifier with linear kernel is employed in comparison to other classifiers, as shown in Tables 3 and 4. Furthermore, it should be mentioned that the K-NN classifier performs better only in feature measures. Additionally, Table 4 results show that the proposed segmentation improves the performance of the brain tumor detection system over the alternative system. The outcomes of the proposed GMM-LA and BMM-LA segmentation methods-based brain tumor detection system are shown in Table 5. As can be observed, a brain tumor detection system using the HCNN-RF segmentation method performs significantly better in terms of accuracy, specificity, and sensitivity than GMM-LA and BMM-LA segmentation. The results indicate that using the suggested method for picture segmentation performs better than GMM and BMM because of HCNN's versatility. A receiver operating characteristic (ROC) curve is a graphical representation of a binary classifier system's diagnostic capacity as represented by its discrimination threshold. Plotting the TP rate against the FP rate at various threshold settings results in the ROC curve. Figure 9 displays the ROC curves for the segmentation-based brain tumor detection systems using BMM-LA, HCNN-RF, and GMM-LA.

**Table 5:** The evaluation results of the brain tumor diagnosis system with GMM-LA and BMM-LA segmentation.

Classifier	Measures	Threefold (%)	Fivefold (%)	Ninefold (%)
SVM (kernel linear)	Accuracy	89.44	89.48	92.04
	Specificity	92.9	92.78	93.07
	Sensitivity	86.27	86.74	91.64
	F1 score	85.40	99.25	97.40
SVM (kernel polynomial)	Accuracy	84.41	81.76	78.01
	Specificity	86.81	84.34	72.38
	Sensitivity	82.4	78.62	82.33
	F1 score	98.20	100	88.60
SVM (kernel RBF)	Accuracy	85.7	88.15	89.44
	Specificity	74.12	78.35	79.42
	Sensitivity	96.09	96.41	98.06
	F1 score	80.63	86.30	99.30
Decision tree	Accuracy	89.38	88.21	92.07
	Specificity	90.03	93.36	90.9
	Sensitivity	88.21	83.89	94.06
	F1 score	90.60	89.20	100
*K*-NN (*K* = 1)	Accuracy	89.1	91.15	89.41
	Specificity	72.52	86.61	89.51
	Sensitivity	100.86	95.18	88.82
	F1 score	85.10	92.40	90.20
*K*-NN (*K* = 4)	Accuracy	94.11	91.16	94.46
	Specificity	85.86	85.95	90.52
	Sensitivity	100.86	95.23	98.64
	F1 score	86.31	89.60	94.50

**Figure 10:**
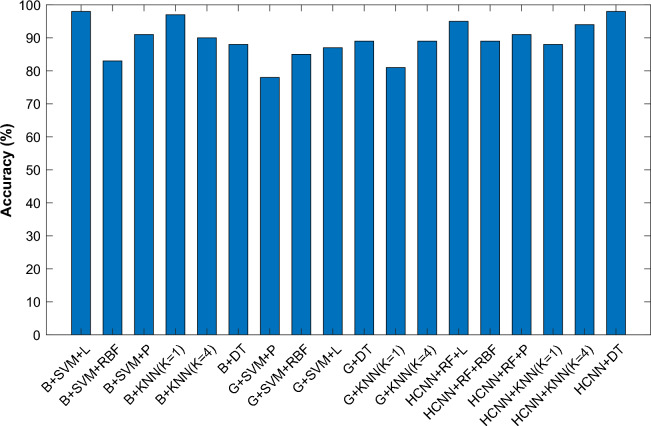
Accuracy of brain tumor diagnosis as determined by several approaches.

Figure 10 displays the evaluation outcomes for several modalities. As can be observed, the suggested HCNN-RF segmentation works best when combined with an SVM with a linear kernel as a classifier because it can precisely identify the tumor's location and hence enhances the system's accuracy.

## Conclusion

According to the research conducted and the findings of the proposed method for diagnosing and identifying brain tumors in MRI images, it can be concluded that the HCNN-RF method, utilizing convolutional neural networks and random forest classification algorithms, has proven to be effective in providing high accuracy in diagnosing brain tumors. This method initially employs pre-processing techniques to enhance the quality of MRI images, followed by the application of the watershed algorithm along with a combination of convolutional neural networks and random forest classification to identify and classify tumors. Evaluations have demonstrated that this approach significantly enhances accuracy, sensitivity, and the F1 score compared to alternative methods, with an increase of 99.3% in accuracy, 97.3% in sensitivity, and 98.5% in the F1 score, respectively. Consequently, this research underscores the substantial capability of our proposed method in recognizing and categorizing MRI images, thereby serving as a reliable system for diagnosing brain tumors.

According to the evaluations made to improve the proposed method, future works can include the following:

- Improving the performance of the method by using deeper neural networks: Using deeper and more complex neural networks can provide a significant improvement in the accuracy and performance of the proposed method.
- Using new pre-processing methods: Using pre-processing methods based on deep learning and machine learning can improve the quality and accuracy of brain tumor diagnosis.
- Development of the method to detect tumors in other parts of the body: The expansion of the proposed method to detect tumors in other parts of the body, such as the detection of cancer in other organs and organs, can provide more applications for this method.

## Data Availability

In the proposed work, we have used a dataset of 74 MR images collected from Cancer Imaging Achievements (TCIA) (https://imaging.cancer.gov/informatics/cancer_imaging_archive.htm) and Harvard Medical School. This data set includes 41 images with tumor (glioblastoma type) and 33 normal images. Also, if anyone wants to request data from this study, they can contact the corresponding author.
